# Poly[[tetra­aquadi-μ_3_-oxalato-μ_2_-oxalato-diprasedymium(III)] dihydrate]

**DOI:** 10.1107/S1600536809053598

**Published:** 2009-12-19

**Authors:** Jian-Hong Chen, Hua-Cai Fang, Hong-Yang Jia, Shan-Shan Li, Yue-Peng Cai

**Affiliations:** a School of Chemistry and Environment, South China Normal University, Guangzhou 510631, People’s Republic of China

## Abstract

In the title compound, {[Pr_2_(C_2_O_4_)_3_(H_2_O)_4_]·2H_2_O}_*n*_, the three-dimensional network structure has the Pr^III^ ion coordinated by nine O atoms in a distorted tricapped trigonal-prismatic geometry. The coordinated and uncoordinated water mol­ecules inter­act with the carboxyl­ate O atoms to consolidate the network *via* O—H⋯O hydrogen bonds.

## Related literature

For general background, see: Benson *et al.* (2000 ▶).
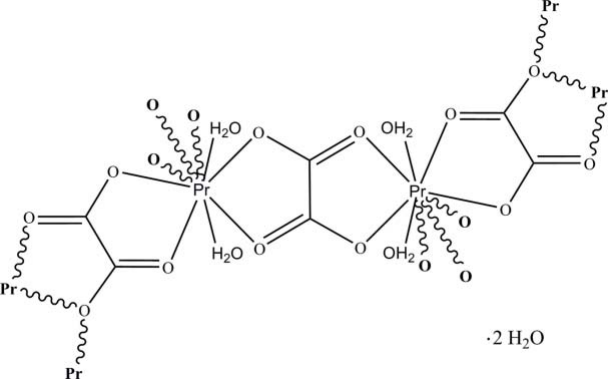


## Experimental

### 

#### Crystal data

[Pr_2_(C_2_O_4_)_3_(H_2_O)_4_]·2H_2_O
*M* *_r_* = 653.98
Monoclinic, 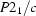

*a* = 9.8834 (5) Å
*b* = 8.2811 (4) Å
*c* = 10.1818 (5) Å
β = 99.053 (1)°
*V* = 822.95 (7) Å^3^
*Z* = 2
Mo *K*α radiation
μ = 5.95 mm^−1^
*T* = 298 K
0.26 × 0.22 × 0.16 mm

#### Data collection

Bruker SMART diffractometer
Absorption correction: multi-scan (*SADABS*; Sheldrick, 1996 ▶) *T*_min_ = 0.307, *T*_max_ = 0.450
4111 measured reflections
1487 independent reflections
1397 reflections with *I* > 2σ(*I*)
*R*_int_ = 0.023

#### Refinement

*R*[*F* ^2^ > 2σ(*F* ^2^)] = 0.016
*wR*(*F* ^2^) = 0.035
*S* = 1.07
1487 reflections
139 parameters
9 restraints
H atoms treated by a mixture of independent and constrained refinement
Δρ_max_ = 0.44 e Å^−3^
Δρ_min_ = −0.54 e Å^−3^

### 

Data collection: *SMART* (Bruker, 1998 ▶); cell refinement: *SAINT* (Bruker, 1999 ▶); data reduction: *SAINT*; program(s) used to solve structure: *SHELXS97* (Sheldrick, 2008 ▶); program(s) used to refine structure: *SHELXL97* (Sheldrick, 2008 ▶); molecular graphics: *SHELXTL* (Sheldrick, 2008 ▶); software used to prepare material for publication: *SHELXTL*.

## Supplementary Material

Crystal structure: contains datablocks I, global. DOI: 10.1107/S1600536809053598/ng2682sup1.cif

Structure factors: contains datablocks I. DOI: 10.1107/S1600536809053598/ng2682Isup2.hkl

Additional supplementary materials:  crystallographic information; 3D view; checkCIF report

## Figures and Tables

**Table 1:** Hydrogen-bond geometry (Å, °)

*D*—H⋯*A*	*D*—H	H⋯*A*	*D*⋯*A*	*D*—H⋯*A*
O7—H7*A*⋯O9^i^	0.85 (1)	1.89 (1)	2.732 (3)	171 (3)
O7—H7*B*⋯O1^ii^	0.85 (1)	1.94 (2)	2.732 (3)	155 (4)
O8—H8*A*⋯O2^iii^	0.84 (1)	2.06 (1)	2.900 (3)	175 (4)
O8—H8*A*⋯O3^iv^	0.84 (1)	2.59 (4)	3.026 (3)	114 (3)
O8—H8*B*⋯O9^v^	0.84 (1)	2.12 (2)	2.949 (4)	167 (6)
O9—H9*B*⋯O6^vi^	0.83 (1)	1.99 (1)	2.820 (3)	178 (4)
O9—H9*A*⋯O8	0.84 (1)	2.05 (1)	2.881 (4)	173 (4)

Symmetry codes: (i) 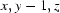; (ii) 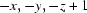; (iii) 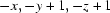; (iv) 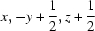; (v) 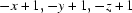; (vi) 
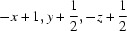
.

**Fig. 1.**
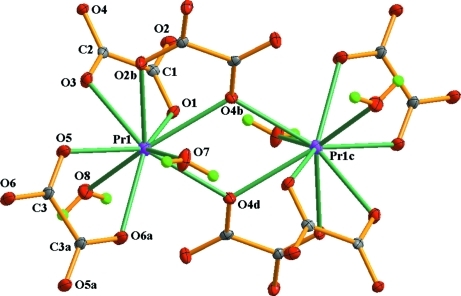
The structure of the title compound (I) with 30% probability ellipsoids. Symmetry code a: 1 - x,-y,1 - z; b: -x,-1/2 + y,0.5 - z; c: -x,-y,1 - z; d: x,0.5 - y,1/2 + z.

**Fig. 2.**
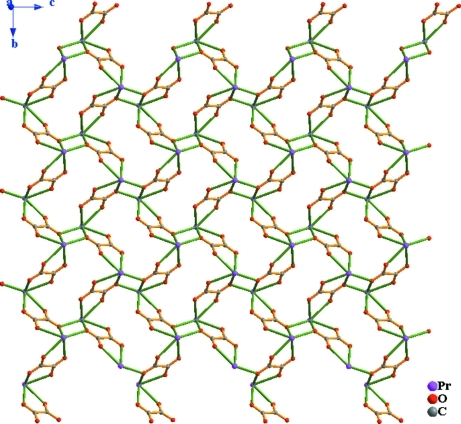
A view of one-dimensional chain constructed by hydrogen bonding interactions. Symmetry code a: x - 1,y,z.

## supplementary crystallographic information

### Comment

Though complexes including oxalato and praseodymium(III) have been extensively investigated, the crystal structure of praseodymium oxalate with one-dimensional, two-dimensional and three-dimensional topologies and different solvent molecules have constantly being reported recently. In this paper, we would like to report the synthesis and crystal structure of a three-dimensional network complex including oxalato and praseodymium(III) with one lattice water molecule per unsymmetrical unit. The hydrothermal reaction of Pr_6_O_11_ and oxalic acid in H_2_O afforded the Pr^III^ title polymeric complex, [(Pr(C_2_O_4_)_1.5_(H_2_O)_2_).H_2_O]_n_. The Pr^III^ ion is coordinated by nine O atoms from two water molecules and four carboxylate groups of oxalate ligands in an irregular coordination geometry (Figure 1). Two oxalate groups of the oxalate anions bridge two symmetry-related Pr^III^ atoms, giving rise to a layer-like structure extending along [100] (Figure 2). These parallel layers are further connected *via* chelating coordination of oxalate anions into a three-dimensional network (Figure 3) along [010] plane. Moreover, the coordinated/non-coordinated water molecules interact with the carboxylate oxygen atoms from the layers *via* O—H···O hydrogen bonds (Table 1), which contributes to the additional stability of the structure.

### Experimental

A suspension of praseodymium oxide (205 mg, 0.20 mmol) in in water (20 ml) was slowly added to a solution of oxalic acid (0.10 mmol) in water (10 ml). The resultant mixture was sealed in a 50 cm^3^ stainless steel reactor with Teflon liner and kept under autogenous pressure at 100 ° for 78 h, and then cooled to room temperature at a rate of 0.5 °. min^-1^. Colorless block crystals of the compound suited for single-crystal X-ray diffraction analyses formed with a yield of approximate 65%. The assigned structure was substantiated by elemental analysis; calculated for C_3_H_6_O_9_Pr: C 11.01, H 1.83%; found: C 10.96, H 1.95%.

### Refinement

The structure was solved using direct methods followed by Fourier synthesis. Non-H atoms were refined anisotropically. The water H atoms were located in a difference Fourier map, and were refined with distance restraints of O—H = 0.84(0.01) and H···H 1.428 (0.002) |%A, but their *U*_iso_ values were set equal to 1.5 *U*_eq_(parent atom O).

### Crystal data

**Table:**

[Pr_2_(C_2_O_4_)_3_(H_2_O)_4_]·2H_2_O	*F*(000) = 620
*M**_r_* = 653.98	*D*_x_ = 2.639 Mg m^−^^3^
Monoclinic, *P*2_1_/*c*	Mo *K*α radiation, λ = 0.71073 Å
*a* = 9.8834 (5) Å	Cell parameters from 3328 reflections
*b* = 8.2811 (4) Å	θ = 3.2–28.6°
*c* = 10.1818 (5) Å	µ = 5.95 mm^−^^1^
β = 99.053 (1)°	*T* = 298 K
*V* = 822.95 (7) Å^3^	Block, yellow
*Z* = 2	0.26 × 0.22 × 0.16 mm

### Data collection

**Table:**

Bruker SMART diffractometer	1487 independent reflections
Radiation source: fine-focus sealed tube	1397 reflections with *I* > 2σ(*I*)
graphite	*R*_int_ = 0.023
φ and ω scans	θ_max_ = 25.3°, θ_min_ = 2.1°
Absorption correction: multi-scan (*SADABS*; Sheldrick, 1996)	*h* = −11→11
*T*_min_ = 0.307, *T*_max_ = 0.450	*k* = −9→9
4111 measured reflections	*l* = −12→7

### Refinement

**Table:**

Refinement on *F*^2^	Secondary atom site location: difference Fourier map
Least-squares matrix: full	Hydrogen site location: inferred from neighbouring sites
*R*[*F*^2^ > 2σ(*F*^2^)] = 0.016	H atoms treated by a mixture of independent and constrained refinement
*wR*(*F*^2^) = 0.035	*w* = 1/[σ^2^(*F*_o_^2^) + (0.0087*P*)^2^ + 0.7364*P*] where *P* = (*F*_o_^2^ + 2*F*_c_^2^)/3
*S* = 1.07	(Δ/σ)_max_ = 0.007
1487 reflections	Δρ_max_ = 0.44 e Å^−^^3^
139 parameters	Δρ_min_ = −0.54 e Å^−^^3^
9 restraints	Extinction correction: *SHELXL97* (Sheldrick, 2008), Fc^*^=kFc[1+0.001xFc^2^λ^3^/sin(2θ)]^-1/4^
Primary atom site location: structure-invariant direct methods	Extinction coefficient: 0.0127 (4)

### Special details

**Table:**

Geometry. All e.s.d.'s (except the e.s.d. in the dihedral angle between two l.s. planes)are estimated using the full covariance matrix. The cell e.s.d.'s are takeninto account individually in the estimation of e.s.d.'s in distances, anglesand torsion angles; correlations between e.s.d.'s in cell parameters are onlyused when they are defined by crystal symmetry. An approximate (isotropic)treatment of cell e.s.d.'s is used for estimating e.s.d.'s involving l.s. planes.
Refinement. Refinement of *F*^2^ against ALL reflections. The weighted *R*-factor *wR* andgoodness of fit *S* are based on *F*^2^, conventional *R*-factors *R* are basedon *F*, with *F* set to zero for negative *F*^2^. The threshold expression of*F*^2^ > σ(*F*^2^) is used only for calculating *R*-factors(gt) *etc*. and isnot relevant to the choice of reflections for refinement. *R*-factors basedon *F*^2^ are statistically about twice as large as those based on *F*, and *R*-factors based on ALL data will be even larger.

### Fractional atomic coordinates and isotropic or equivalent isotropic displacement parameters (Å^2^)

**Table:**

	*x*	*y*	*z*	*U*_iso_*/*U*_eq_	
Pr1	0.185002 (15)	0.098389 (18)	0.437068 (15)	0.01483 (9)	
O1	0.0042 (2)	0.3167 (2)	0.4302 (2)	0.0210 (5)	
O2	−0.1229 (2)	0.4945 (3)	0.2991 (2)	0.0249 (5)	
O3	0.1838 (2)	0.3092 (3)	0.2610 (2)	0.0229 (5)	
O4	0.0564 (2)	0.4802 (3)	0.12614 (19)	0.0196 (5)	
O5	0.4011 (2)	0.0665 (3)	0.3529 (2)	0.0275 (5)	
O6	0.6199 (2)	−0.0090 (3)	0.3924 (2)	0.0287 (5)	
O7	0.1928 (2)	−0.2047 (3)	0.4324 (3)	0.0357 (6)	
H7A	0.255 (3)	−0.260 (3)	0.406 (4)	0.054*	
H7B	0.153 (3)	−0.250 (4)	0.490 (3)	0.054*	
O8	0.3072 (3)	0.3550 (3)	0.5421 (2)	0.0296 (5)	
H8A	0.252 (3)	0.393 (4)	0.589 (3)	0.044*	
O9	0.3963 (3)	0.5983 (3)	0.3724 (3)	0.0353 (6)	
H9B	0.393 (4)	0.566 (5)	0.2945 (16)	0.053*	
C1	−0.0222 (3)	0.4050 (3)	0.3291 (3)	0.0172 (6)	
C2	0.0829 (3)	0.3968 (3)	0.2319 (3)	0.0162 (6)	
C3	0.5054 (3)	0.0165 (4)	0.4261 (3)	0.0200 (7)	
H8B	0.3907 (13)	0.358 (8)	0.576 (4)	0.13 (3)*	
H9A	0.370 (7)	0.534 (5)	0.427 (3)	0.13 (3)*	

### Atomic displacement parameters (Å^2^)

**Table:**

	*U*^11^	*U*^22^	*U*^33^	*U*^12^	*U*^13^	*U*^23^
Pr1	0.01116 (11)	0.01772 (12)	0.01591 (11)	0.00145 (6)	0.00305 (7)	0.00222 (6)
O1	0.0236 (11)	0.0229 (11)	0.0185 (11)	0.0044 (9)	0.0094 (9)	0.0063 (9)
O2	0.0248 (12)	0.0312 (12)	0.0191 (11)	0.0126 (10)	0.0051 (10)	0.0018 (9)
O3	0.0186 (11)	0.0244 (11)	0.0274 (12)	0.0049 (9)	0.0090 (10)	0.0067 (10)
O4	0.0179 (11)	0.0261 (11)	0.0149 (11)	0.0013 (9)	0.0033 (9)	0.0049 (9)
O5	0.0183 (12)	0.0451 (14)	0.0198 (12)	0.0077 (10)	0.0052 (10)	0.0090 (10)
O6	0.0195 (12)	0.0478 (14)	0.0204 (12)	0.0121 (10)	0.0078 (10)	0.0095 (11)
O7	0.0351 (14)	0.0252 (13)	0.0533 (17)	0.0080 (11)	0.0273 (13)	0.0110 (11)
O8	0.0303 (14)	0.0317 (13)	0.0272 (13)	−0.0058 (11)	0.0057 (11)	−0.0034 (10)
O9	0.0397 (15)	0.0410 (15)	0.0274 (14)	0.0031 (12)	0.0118 (13)	−0.0034 (12)
C1	0.0182 (16)	0.0162 (14)	0.0170 (15)	−0.0001 (12)	0.0023 (13)	−0.0043 (12)
C2	0.0188 (16)	0.0156 (14)	0.0141 (15)	−0.0066 (12)	0.0022 (12)	−0.0034 (12)
C3	0.0170 (16)	0.0257 (16)	0.0183 (16)	0.0030 (13)	0.0064 (13)	0.0042 (13)

### Geometric parameters (Å, °)

**Table:**

Pr1—O5	2.438 (2)	O4—Pr1^v^	2.552 (2)
Pr1—O6^i^	2.495 (2)	O4—Pr1^iv^	2.566 (2)
Pr1—O3	2.501 (2)	O5—C3	1.244 (4)
Pr1—O7	2.512 (2)	O6—C3	1.251 (4)
Pr1—O1	2.535 (2)	O6—Pr1^i^	2.495 (2)
Pr1—O2^ii^	2.537 (2)	O7—H7A	0.847 (10)
Pr1—O4^iii^	2.552 (2)	O7—H7B	0.845 (10)
Pr1—O4^ii^	2.566 (2)	O8—H8A	0.842 (10)
Pr1—O8	2.590 (2)	O8—H8B	0.844 (10)
O1—C1	1.257 (4)	O9—H9B	0.832 (10)
O2—C1	1.240 (4)	O9—H9A	0.839 (10)
O2—Pr1^iv^	2.537 (2)	C1—C2	1.545 (4)
O3—C2	1.231 (4)	C3—C3^i^	1.549 (6)
O4—C2	1.271 (4)		
			
O5—Pr1—O6^i^	65.72 (7)	O3—Pr1—O8	70.89 (8)
O5—Pr1—O3	74.28 (7)	O7—Pr1—O8	144.46 (8)
O6^i^—Pr1—O3	128.45 (7)	O1—Pr1—O8	72.97 (8)
O5—Pr1—O7	81.61 (7)	O2^ii^—Pr1—O8	134.18 (7)
O6^i^—Pr1—O7	72.19 (8)	O4^iii^—Pr1—O8	98.79 (7)
O3—Pr1—O7	132.90 (8)	O4^ii^—Pr1—O8	140.47 (7)
O5—Pr1—O1	136.09 (7)	C1—O1—Pr1	119.53 (18)
O6^i^—Pr1—O1	134.01 (7)	C1—O2—Pr1^iv^	120.07 (19)
O3—Pr1—O1	63.82 (6)	C2—O3—Pr1	119.75 (18)
O7—Pr1—O1	137.35 (7)	C2—O4—Pr1^v^	116.09 (18)
O5—Pr1—O2^ii^	74.00 (7)	C2—O4—Pr1^iv^	118.63 (18)
O6^i^—Pr1—O2^ii^	127.17 (7)	Pr1^v^—O4—Pr1^iv^	117.51 (8)
O3—Pr1—O2^ii^	65.42 (7)	C3—O5—Pr1	120.94 (18)
O7—Pr1—O2^ii^	69.33 (8)	C3—O6—Pr1^i^	119.5 (2)
O1—Pr1—O2^ii^	98.71 (7)	Pr1—O7—H7A	125 (2)
O5—Pr1—O4^iii^	143.64 (7)	Pr1—O7—H7B	114 (2)
O6^i^—Pr1—O4^iii^	79.71 (7)	H7A—O7—H7B	115.0 (15)
O3—Pr1—O4^iii^	140.53 (7)	Pr1—O8—H8A	104 (2)
O7—Pr1—O4^iii^	77.27 (7)	Pr1—O8—H8B	124 (4)
O1—Pr1—O4^iii^	76.71 (6)	H8A—O8—H8B	115.7 (15)
O2^ii^—Pr1—O4^iii^	123.63 (6)	H9B—O9—H9A	117.4 (16)
O5—Pr1—O4^ii^	134.60 (7)	O2—C1—O1	127.3 (3)
O6^i^—Pr1—O4^ii^	130.37 (7)	O2—C1—C2	117.6 (3)
O3—Pr1—O4^ii^	100.79 (7)	O1—C1—C2	115.1 (3)
O7—Pr1—O4^ii^	69.15 (7)	O3—C2—O4	125.1 (3)
O1—Pr1—O4^ii^	68.99 (7)	O3—C2—C1	118.2 (3)
O2^ii^—Pr1—O4^ii^	63.66 (6)	O4—C2—C1	116.7 (3)
O4^iii^—Pr1—O4^ii^	62.49 (8)	O5—C3—O6	126.3 (3)
O5—Pr1—O8	81.77 (8)	O5—C3—C3^i^	117.6 (3)
O6^i^—Pr1—O8	72.37 (8)	O6—C3—C3^i^	116.1 (3)

Symmetry codes: (i) −*x*+1, −*y*, −*z*+1; (ii) −*x*, *y*−1/2, −*z*+1/2; (iii) *x*, −*y*+1/2, *z*+1/2; (iv) −*x*, *y*+1/2, −*z*+1/2; (v) *x*, −*y*+1/2, *z*−1/2.

### Hydrogen-bond geometry (Å, °)

**Table:**

*D*—H···*A*	*D*—H	H···*A*	*D*···*A*	*D*—H···*A*
O7—H7A···O9^vi^	0.85 (1)	1.89 (1)	2.732 (3)	171 (3)
O7—H7B···O1^vii^	0.85 (1)	1.94 (2)	2.732 (3)	155 (4)
O8—H8A···O2^viii^	0.84 (1)	2.06 (1)	2.900 (3)	175 (4)
O8—H8A···O3^iii^	0.84 (1)	2.59 (4)	3.026 (3)	114 (3)
O8—H8B···O9^ix^	0.84 (1)	2.12 (2)	2.949 (4)	167 (6)
O9—H9B···O6^x^	0.83 (1)	1.99 (1)	2.820 (3)	178 (4)
O9—H9A···O8	0.84 (1)	2.05 (1)	2.881 (4)	173 (4)

Symmetry codes: (vi) *x*, *y*−1, *z*; (vii) −*x*, −*y*, −*z*+1; (viii) −*x*, −*y*+1, −*z*+1; (iii) *x*, −*y*+1/2, *z*+1/2; (ix) −*x*+1, −*y*+1, −*z*+1; (x) −*x*+1, *y*+1/2, −*z*+1/2.
